# Harmonic radar tracking reveals random dispersal pattern of bumblebee (*Bombus terrestris*) queens after hibernation

**DOI:** 10.1038/s41598-019-40355-6

**Published:** 2019-03-20

**Authors:** James C. Makinson, Joseph L. Woodgate, Andy Reynolds, Elizabeth A. Capaldi, Clint J. Perry, Lars Chittka

**Affiliations:** 1 School of Biological and Chemical Sciences, Queen Mary University of London, London, E1 4NS UK; 2 Hawkesbury Institute for the Environment, Western Sydney University, Penrith, New South Wales Australia; 3 Rothamsted Research, Harpenden, AL5 2JQ UK; 4 Department of Biology, Bucknell University, Lewisburg, Pennsylvania USA; 5 Wissenschaftskolleg, Institute for Advanced Study, D19413 Berlin, Germany

## Abstract

The dispersal of animals from their birth place has profound effects on the immediate survival and longer-term persistence of populations. Molecular studies have estimated that bumblebee colonies can be established many kilometers from their queens’ natal nest site. However, little is known about when and how queens disperse during their lifespan. One possible life stage when dispersal may occur, is directly after emerging from hibernation. Here, harmonic radar tracking of artificially over-wintered *Bombus terrestris* queens shows that they spend most of their time resting on the ground with intermittent very short flights (duration and distance). We corroborate these behaviors with observations of wild queen bees, which show similar prolonged resting periods between short flights, indicating that the behavior of our radar-monitored bees was not due to the attachment of transponders nor an artifact of the bees being commercially reared. Radar-monitored flights were not continuously directed away from the origin, suggesting that bees were not intentionally trying to disperse from their artificial emergence site. Flights did not loop back to the origin suggesting bees were not trying to remember or get back to the original release site. Most individuals dispersed from the range of the harmonic radar within less than two days and did not return. Flight directions were not different from a uniform distribution and flight lengths followed an exponential distribution, both suggesting random dispersal. A random walk model based on our observed data estimates a positive net dispersal from the origin over many flights, indicating a biased random dispersal, and estimates the net displacement of queens to be within the range of those estimated in genetic studies. We suggest that a distinct post-hibernation life history stage consisting mostly of rest with intermittent short flights and infrequent foraging fulfils the dual purpose of ovary development and dispersal prior to nest searching.

## Introduction

Wild pollinators are under threat by anthropogenic changes in landscape, including agricultural development and urban sprawl^1^. Knowledge of a species’ dispersal patterns can be crucial in predicting how animals respond to environmental change^2^. Bumblebees are important for the pollination of many native and wild plants throughout temperate ecosystems^3–6^, but the dispersal behaviors of bumblebee queens after hibernation is relatively unknown. Bumblebee colonies are comprised of a queen and up to a few hundred daughter workers. At the end of the colony’s annual life cycle, new queens and males are produced which then leave the hive to mate. The males die before the winter, whereas the new queens hibernate and go on to set up colonies in the spring.

Dispersal of new queen bumblebees from their natal nest sites could occur at various stages of life, including before mating, between mating and hibernation, after emergence from hibernation but before nest searching, or while nest searching. To our knowledge, only two studies have explicitly examined the dispersal of queen bumblebees. By looking at genetic relatedness of bees from nearby colonies, Lepais and colleagues^7^ estimated that new colonies were founded between three and five kilometers from their queen’s natal nest. Another study using genetic analyses and geographical sampling investigated the invasion of imported bumblebee species into Chile and suggested that *Bombus terrestris* queens spread up to about 200 km each year^8^. However, this surprisingly long distance was likely, as the authors point out, aided by the prevailing strong winds across the Andes, and perhaps due to anthropogenic causes, e.g. unintentional automotive transportation of queens within plants or soil.

In temperate regions, bumblebees take an estimated two to three weeks to locate a new nest site and begin to forage for pollen^9^, but there is very limited information on the behavior of queen bumblebees immediately after emergence from hibernation. Several accounts from almost 60 years ago claim that when queen bumblebees first appear in the early days of spring they spend most of their time actively flying and foraging for nectar and pollen, which helps replenish their fat reserves and develop their ovaries, and within a short time they begin searching for nest sites^10–13^. However, there are no empirical data specifically on this life stage. The behavior of newly emerged queens and the contribution of this time period to colony dispersal is unknown. Using harmonic radar tracking of artificially over-wintered queen bees, we describe their behavior upon emergence and use a random walk model to estimate their dispersal pattern and displacement.

## Results

### Radar tracking of artificially over-wintered bumblebee queens

**Figure 1:**
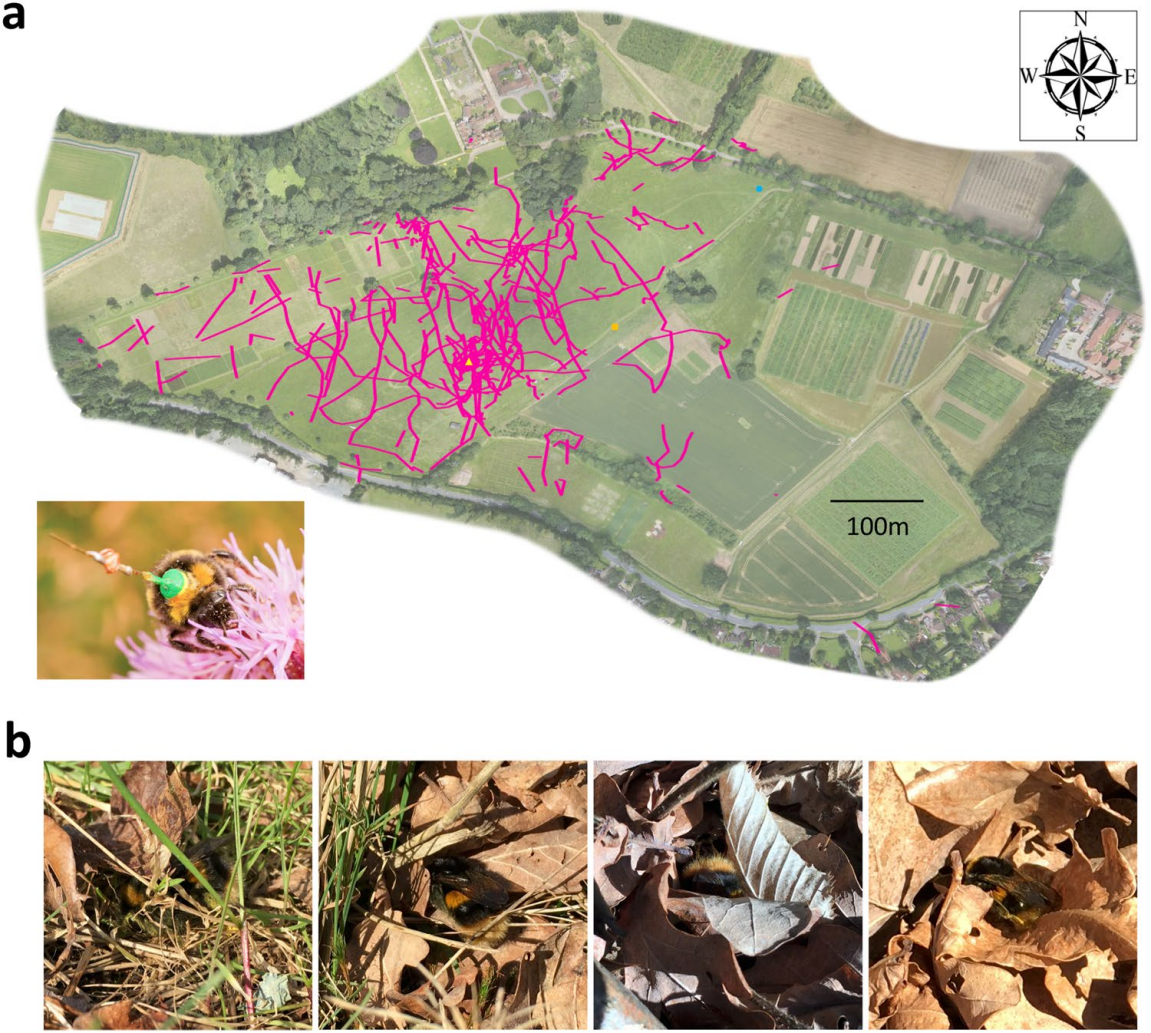
Queen bumblebees (*Bombus terrestris*) that have recently emerged from artificial or natural hibernation spend more time resting than flying. (**a**) Aerial view of field site where harmonic radar tracking took place (an orthomosaic created from drone photography; flight tracks overlaid using MATLAB 2015b). Yellow triangle designates position where queen bumblebees were placed prior to the start of experiment (release site). Orange square and blue circle indicate positions of the harmonic radar for the 17 group- and three individually-monitored bees, respectively. Pink lines indicate flight paths of radar tracked bees. Inset photo: queen bumblebee with a transponder fixed to her thorax. (**b**) Photos show observed behavior of recently emerged queen bumblebees. Most bees we observed landed on the ground after a short flight and proceeded to walk around within a few centimeter radius for several seconds before becoming motionless or, more often, burrowing their body or head into or under vegetation.

We monitored 20 artificially over-wintered queen bumblebees with harmonic radar (Fig. 1a). Queen bumblebees were kept at 4 °C until the morning of the start of experiments (Methods), at which point a transponder was superglued to the thorax of each bee (Fig. 1a inset). Subsequently, each bee was placed in a small depression in the center of a mound of earth (approx. 5 cm high and 15 cm in diameter), where they were allowed to warm up naturally. The activity of all bees was monitored with the harmonic radar for between 4 and 15 hours each day (depending on weather and therefore likelihood of any flights occurring) and until all the bees had permanently dispersed outside the range of the radar (Fig. 1a). Three bees were monitored individually for up to three days each. The other 17 bees were monitored simultaneously for a period of five days.

The harmonic radar tracks of all 20 bumblebees show that queens tended to make very short (duration and distance) flights (means ± s.e. given throughout the text; 14.2 ± 1.0 s, 34.2 ± 2.3 m, n = 264) separated by long periods of inactivity (29.0 ± 13.8 min, n = 11). This suggests that newly emerged queen bumblebees, prior to nest searching, may spend less than two percent of their day flying, while spending a large majority of their time resting on the ground.

### Post-hibernation behavior of wild bumblebee queens

To ensure that the behavior observed in harmonic radar monitored queen bumblebees was not due to the mass of the transponders attached to them, nor due to the fact that they came from commercially reared colonies, we also observed the behavior of recently emerged wild queen bumblebees (Methods). We found that wild queen bumblebees, recently emerged from hibernation (Methods), also spent long periods of time on the ground (13.3 ± 1.9 min; n = 74) between short (duration and distance) flights (8.7 ± 1.1 s, 4.1 ± 0.7 m; n = 34).

Wild queen bumblebees usually sat very still shortly after landing. Immediately after landing and occasionally between periods of stillness, bees would take a very short, usually circuitous walk (radius approx. 2–5 cm), move under vegetation or groom themselves, and then become motionless. Besides these very short and infrequent movements, the time spent on the ground did not seem to involve any activity other than resting. No feeding or interaction with other queens was observed while on the ground. On only three of our 140 observations, occurring only on the last day of observations, the focal queen was seen to fly close to the ground in a zig-zagging pattern characteristic of nest searching, although we did not observe any of these queens enter or examine a potential nest site. On the first day of observations we noticed large numbers of queen bees feeding on the catkins of a nearby willow tree (*Salix caprea*). Of our focal queens, we observed one bee switch from resting to feeding and two bees switch from feeding on the willow tree to resting on the ground. However, most of the bees we observed resting on the ground did not feed from the nearby tree during our observations. On subsequent days we observed no feeding behavior. Resting behavior of queen bumblebees consisted of, in 61% of the bumblebees observed, positioning their heads under a leaf or within the folds of leaves, and many times crawling under leaves (Fig. 1b). At no point did we observe bees during these between flight periods examining potential nest sites in the ground or burrowing underground.

### Dispersal behavior of artificially over-wintered queens

**Figure 2:**
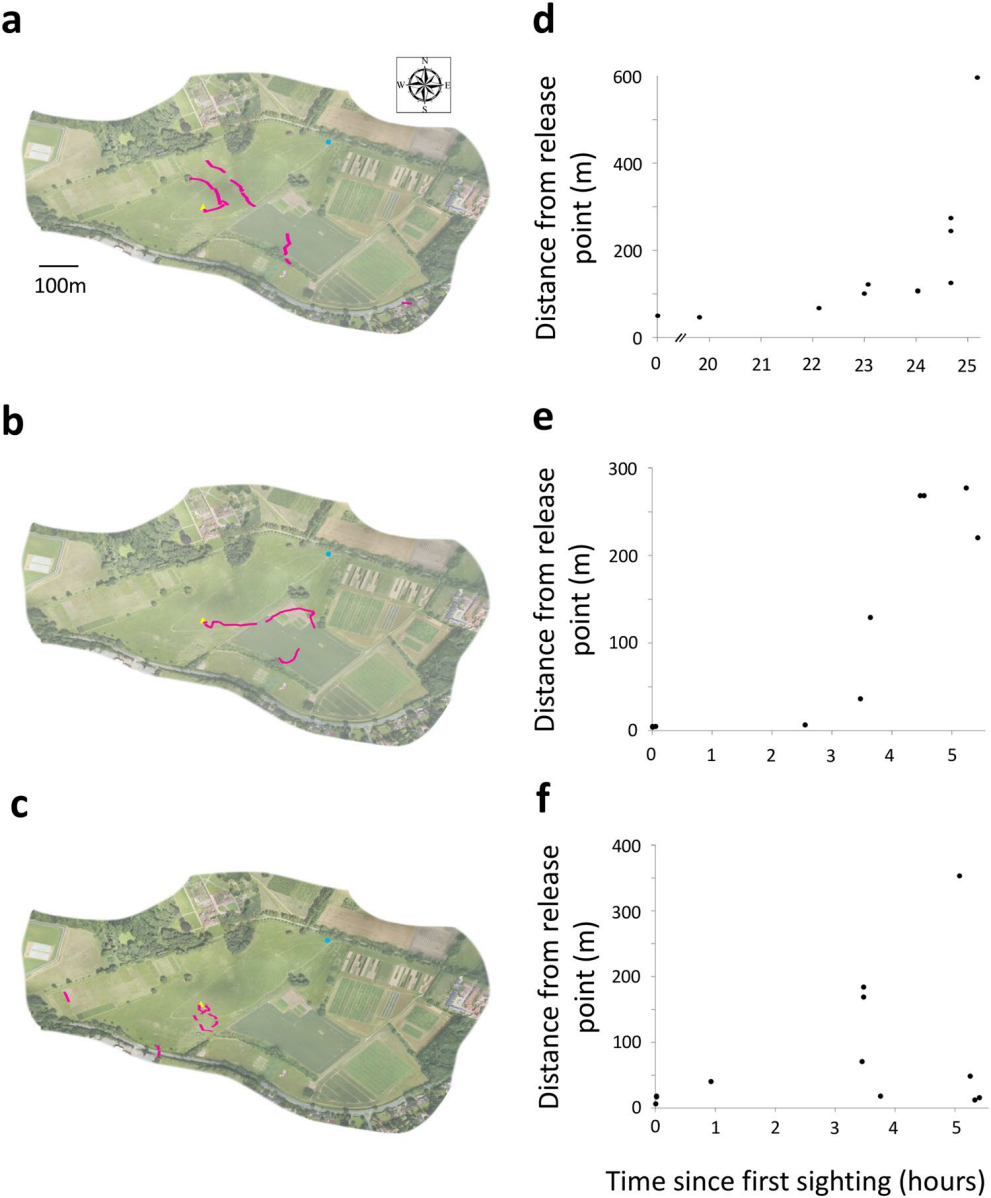
Individually-monitored queen bumblebees disperse slowly from their hibernation site. (**a**–**c**) Aerial view of field site for radar tracking with the flights (pink lines) of three artificially over-wintered queen bumblebees (an orthomosaic created from drone photography; flight tracks overlaid using MATLAB 2015b). Yellow triangle indicates release site. Blue circle indicates position of the harmonic radar. **(e,f)** Euclidean distance from the release point to the last positional fix in each flight of each individually-monitored bee.

Monitoring the activity of single queen bees allowed us to examine individuals’ behavior over time. The three individually-monitored queens varied in their flight behavior, but all showed multiple changes in direction during the day (Fig. 2a–c). Despite not maintaining a consistent flight direction, two bees flew beyond the range of the radar (~600 m within the local topography) on the day of release and the other left the field site within two days, after rain stopped activity on the first day (Fig. 2d–f). None were observed again on subsequent days.

**Figure 3:**
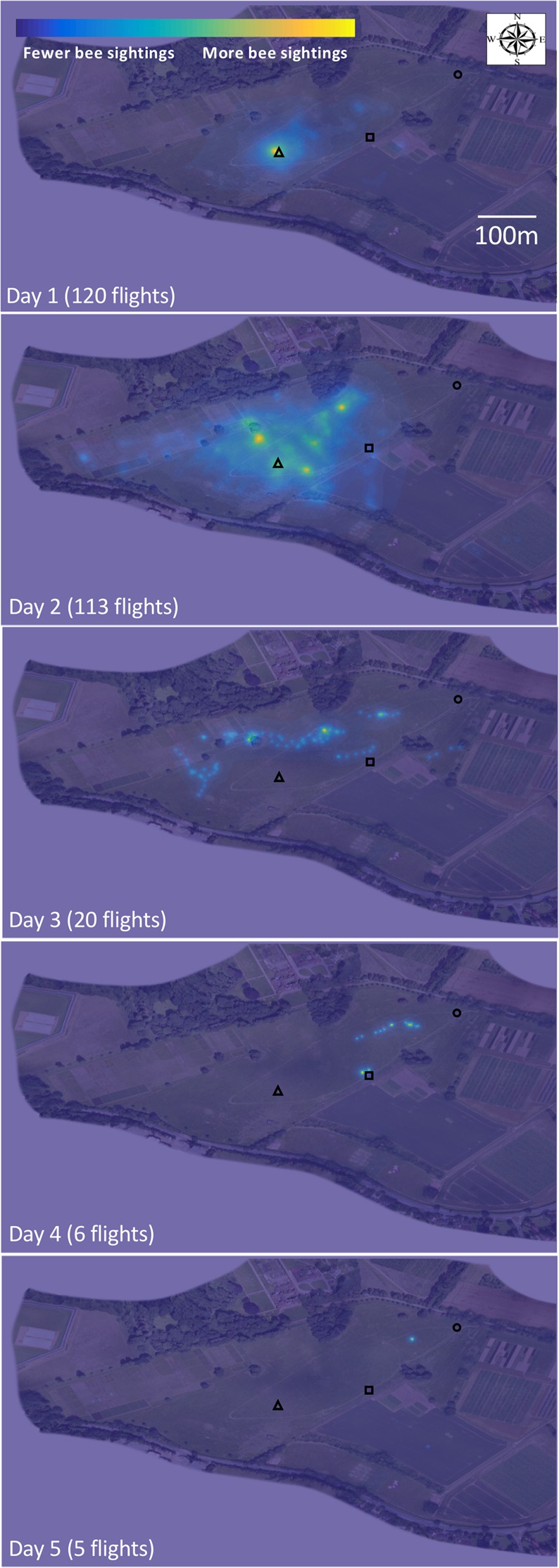
Queen bumblebees disperse from their hibernation site with many short flights between long rest periods. (**a**) Data density plots of bee positional fixes overlaid onto aerial photos (an orthomosaic created from drone photography) of radar tracking field site, monitoring 17 queen bumblebees simultaneously over five days (flight tracks overlaid using MATLAB 2015b). During the first few hours of the first day, most bees remain near the release point, indicated by the high density of positional fixes (yellow). Bees disperse slowly over a day and a half. Near the end of day two, many bees go past the range of the radar indicated by the dramatic drop in positional fixes. Open black triangle indicates position where queen bumblebees were placed into the ground to emerge from prior to experiment. Open black circle indicates position of the harmonic radar.

To get a better understanding of the overall dispersal behavior of queen bees, we monitored a group of 17 simultaneously-released queen bumblebees for five days. By the fifth day, all bees had dispersed beyond the range of the harmonic radar (~600 m; Fig. 3). Because all 17 bees were active during the same period and because we were unable to locate and observe individuals once they had left the release site, it was impossible to identify which bee produced which individual flight. However, the density of positional fixes moved from near the origin to spread across the observable field area within one day (Fig. 3). Further, the number of flights observed decreased dramatically after the second day (Fig. 3). These results indicate that most queens dispersed from the range of the radar within less than two days and did not return.

**Figure 4:**
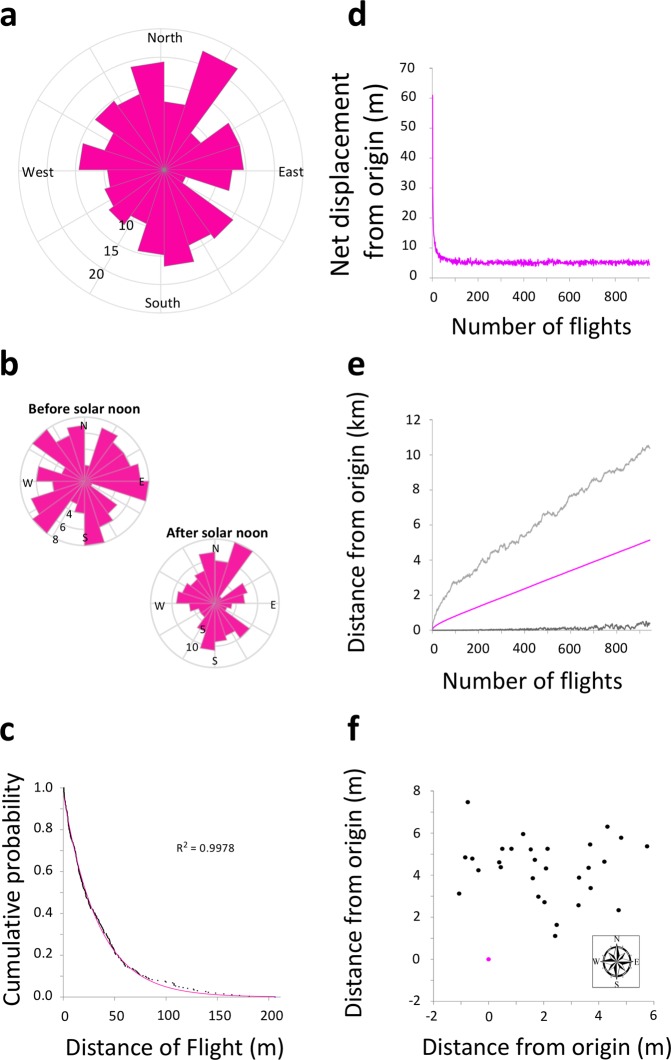
Queen bumblebees slowly disperse from their hibernation site in a random manner. (**a**) Radial histogram of flight directions with cardinal directions indicated. Bees showed no overall directional preference, as the distribution of directions was no different statistically from a uniform distribution. **(b)** Radial histograms show that the position of the sun had no effect on the directional preferences of bees as the distribution of flight directions before and after solar noon was no different from a uniform distribution. **(c)** The cumulative probability of flight distances, calculated using the line connecting the first and last positional fix of each flight, followed an exponential distribution, indicative of a random dispersal (black points). Pink line: cumulative probabilities of an exponential distribution for reference. **(d)** Our random walk model, using the observed flight distances and directions of the harmonic radar monitored bees’ activity combined with the time between flights spent on the ground by our individually radar monitored queen bees, estimates over time an average net dispersal distance per flight from the origin of about five and a half meters. **(e)** Our model estimates an average total net displacement from the origin in three weeks to be approximately three kilometers (pink line; light grey line = maximum estimated distance, dark grey line = minimum estimated distance over 1000 iterations of the model). (**f**) Our model’s estimation of overall displacement of bees from the origin is biased towards northerly directions, but varies a lot around an average displacement (pink dot = origin, black dots = 30 example displacement positions from our model).

The distribution of flight headings was not significantly different from a uniform distribution (Kolmogorov-Smirnov Test for uniformity: p = 0.5925, k = 0.0672, n = 264; Fig. 4a) and this did not change throughout the day, i.e. according to the position of the sun (Kolmogorov-Smirnov Test for uniformity - Before solar noon: p = 0.5757, k = 0.0866, n = 120; After solar noon: p = 0.5651, k = 0.0822, n = 144; Fig. 4b). There was no correlation between flight headings and wind direction (circular correlation coefficient: p = 0.749, r = −0.019, n = 264) and there was no correlation between flight distance and wind speed (circular correlation coefficient: p = 0.786, r = 0.017, n = 264). In addition, the flight distances followed an exponential distribution (R^2^ = 0.9978, n = 264; Fig. 4c), suggesting a random dispersal.

### Modelling post-hibernation dispersal

To assess the general dispersal of queen bumblebees, we created a random walk model which utilized the observed flight distances and directions within the group-monitored data combined with the data on time between flights spent on the ground from our individually-monitored queens (Methods). Because individually monitored, radar-tracked queen bumblebees took, on average, 27.8 ± 24.5 min sojourns between flights of, on average, 13.3 ± 4.1 s, and daylight during the early Spring (March) in England is 12 hours and increases towards the summer, a conservative estimate of flight frequency would be 25 flights per day. Note that here we used only those sequential individual flights that ended and began within 2.5 m (the accuracy of the radar) of each other. For each flight, our random walk model chose a flight heading based on the distribution of the data from our harmonic radar monitored bees (Methods). Flight distance was chosen randomly from within the minimum and maximum flight distances observed near the chosen heading (Methods). Although our bees’ flight directions were no different from a uniform distribution, the very slight bias of flights towards northerly directions (144 of 264 total flights; Fig. 4a) resulted, over many flights, in an overall average net displacement away from the origin. Our model estimates a net displacement from the release point over the first day of flights to be 336.2 ± 5.5 meters (25 flights; averaged over 10,000 iterations; Fig. 4e). The majority of the distance gained on the first day results from the first several flights, as the sign of displacement about the origin becomes more random once the net displacement from the origin is much greater than the maximum flight distance observed. As a result, our model estimates that bees start out displacing themselves on average of about 60 meters from the origin on the first flight, but their net displacement per flight quickly asymptotes towards 5.42 ± 0.08 m per flight (within two days; 50 flights; averaged over 10,000 iterations; Fig. 4d). The net displacement from the origin in three weeks (525 flights) is estimated to be 3043.8 ± 31.7 m (averaged over 10,000 iterations; Fig. 4e). Although the slight bias in the observed flights’ directions manifested itself in our model through a net displacement in northerly directions, there was a large amount of variation around the average displacement from the origin (Fig. 4f).

## Discussion

Characterization of animal movement is vital for efforts dealing with conservation and biological invasions. Here, we used harmonic radar to track the movement of bumblebee queens immediately after emergence from (artificial) hibernation. Strikingly, and contrary to the few observations in prior literature^10–13^, bees spent the majority of their time resting on the ground with infrequent, short (time and distance) flights between. Observations of wild queens very early in the spring corroborate these results and suggest that the behavior of our radar-monitored bees was not due to the transponders or an artifact of being commercially reared. All the wild bees we observed spent most of their time motionless on the ground, sometimes under foliage and only occasionally taking flights for short durations and distances. The differences between these and those from radar-monitored bees is undoubtedly due in part to the fact that we were unable to visually track wild queens for more than about 15 meters (Methods). The purpose of the wild queen observations was to verify that the transponders were not the cause of the long resting/short flight behavior we observed in the radar tracks. Indeed, the wild queen bees spent long periods of time on the ground between short flights, indicating that the similar behavior observed in our radar tracked bees is a common phenomenon amongst bumblebee queens after hibernation. Wild queen bumblebees would have had previous experience where they emerge, inviting the question of whether the lack of experience with the area played a role in the behavior of the commercially reared radar tracked bees. However, wild queen bees showed similar long resting durations and short flight durations and distances, suggesting the behavior observed in radar tracked bees was normal.

**Figure 5:**
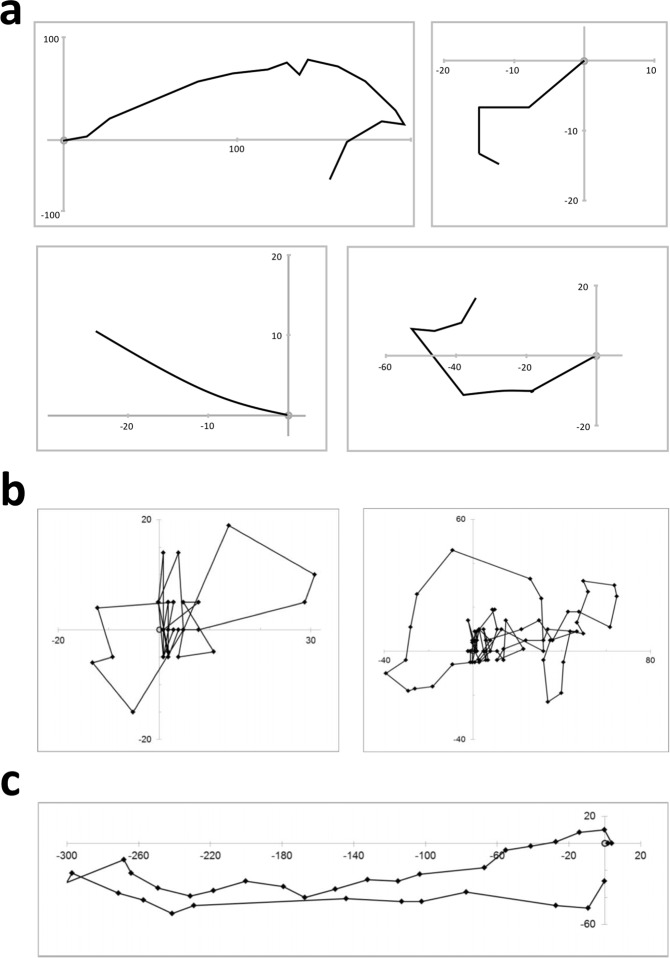
Comparison of bumblebee flight patterns suggest post-hibernation queens disperse in a random manner. (**a**) Flights of four individual radar tracked queen bumblebees in our study. (**b**) Flights of two individual bumblebees on their first flights from the hive. (**c**) Flight of one experienced individual bumblebee on a foraging flight. Closed diamonds indicated positional fixes. Origin is the location of the colony. Axes show distance from the colony in meters. Note that the two orientation flights display loop patterns, while all flights in b and c show returning to the colony, in contrast to flights of post-hibernation queen bees in a and shown in Fig. 1a. Taken from Osborne *et al*.^20^ within the terms of the Creative Commons Attribution License.

Bumblebees, like many other insects, have an internal compass^14^ that allow them to fly long distances in relatively straight lines. Our bees’ flights were certainly not straight and were considerably shorter than the kilometers found to be travelled by bumblebee workers (Fig. 1a)^15,16^. Worker bees make characteristic orientation flights when they first leave the hive, in which they loop near their nest gathering information about their immediate surrounding, enabling them to encode vital information so they can find their way back to the nest later on^17^. However, none of our queen bees’ flights showed the characteristic loop features of an orientation flight (Figs 1a and 5a). Bumblebees are also able to learn and memorize features of their environment (landmarks) in such a manner that they can find their way home after long distance flights or even when being displaced artificially^18^. Our bees did not show any attempt at returning to their place of origin nor did they show any indication that they were consistently avoiding or heading towards local landmarks or prominent features of the horizon profile (Figs 1a and 5b). Together, queen bees’ flight behaviors in this early phase after hibernation resemble a random walk model, similar to Brownian motion^19^.

Lévy flights, a type of random walk suggested to produce optimal search strategies in absence of memory, have been found to characterize many animal movements, including the flight patterns of bumblebees^20^. However, the flight distance distribution of our bees did not show the heavy tail distribution described by a power law, characteristic of Lévy flights, but rather followed an exponential distribution indicative of a random walk^19^ (Fig. 4b).

Vogt and colleagues observed flying, foraging and nest-searching queens and queens with established colonies, in the spring of North Eastern America and Alaska^9^. Their findings suggest that queen bumblebees in temperate regions take on average about three weeks post-emergence (first sighting of queen bees in the early spring) before starting to look for nest sites (zig-zag flight patterns close to the ground and/or investigating holes in the ground and tussocks of vegetation). Once actively searching for a nest, young queens are thought to spend several days or more locating a suitable nest site^12^. Our random walk model, using the distribution of flight directions and distances observed with harmonic radar, estimates that three weeks of the described behavior could displace queen bumblebees, on average, by a distance of approximately three kilometers from their point of emergence. This result is very similar to distances estimated by Lepais and colleagues, measuring genetic relatedness of colonies over multiple seasons, for *Bombus pascuorum* and *Bombus lapidarius* in their natural environment^7^.

Our model is limited by the fact that we are only using five days of harmonic radar data. However, our wild bee observations spanned nine days, and nest searching was seen only on the last day from only three bees. Vogt *et al*.^9^ have shown that bumblebee queens take about three weeks to begin nest searching. This suggests that the observed long resting periods and short flight behavior is sustained over more days and importantly corroborates our harmonic radar observations. Further, our findings corroborate those of Dreier *et al*.^21^ who found that bumblebees exhibit low levels of fine scale spatial genetic structure, i.e. a very low relatedness amongst bee colonies near each other. Their findings would suggest a necessary random and widespread dispersal as seen in the results of Lepais *et al*.^7^ as well as our observations and model.

A slight bias of the observed flights towards northerly directions (144 of 264 total flights) manifested itself within our model as a net displacement from the origin over time, indicating a biased random dispersal. Further experiments will be required to separate potential causes of the slight directional bias, such as the position of the sun within the southern sky, landscape features (e.g. large patches of trees), and wind direction.

Harmonic radar needs a line-of-sight to the transponder in order to detect a signal and cannot reliably detect bees on the ground due to vegetation or undulations of the landscape occluding the transponder. Thus, we could not be sure that between flights, queen bumblebees were not walking, rather than resting. However, wild queen bumblebees spent prolonged periods on the ground and often displayed behavior suggesting that they were resting. During their time on the ground, queens were often observed pushing their heads and body between or under dead leaves or grass (Fig. 1b). Many of the bees we observed displayed a body posture indicating sleep as described in honeybees^22^ where the head sunk downwards and the antennae dropped low and became immobile. Our observations may be indicative of post-hibernation queens having low energy reserves and needing to conserve their fat reserves^10^.

Contrary to textbook accounts based on casual observations^10,12^, our results show that newly emerged queen bumblebees of *Bombus terrestris* spend a large majority of their time resting on the ground between very short flights (tens of seconds) slowly dispersing themselves from their hibernation site. We suggest that the observed behavior may serve the dual purpose of overall dispersal and ovary development. Our results indicate that queen bees are not dispersing immediately and directly away from where they emerged from hibernation, and they are not orienting towards and remaining near their place of origin.

The ability to disperse and the resultant gene flow are important biological factors that help species cope with changes in their environment, like habitat fragmentation and climate change^23^. Land cover changes and habitat fragmentation strongly affect pollinator community composition^24^. Our results and observations suggest practices that may be valuable for conservation efforts concerning bumblebees. For example, if dispersal occurs as a series of very short flights, pollinator friendly corridors between conserved landscape patches could be beneficial. It may also be helpful to leave vegetation, such as leaf litter and long grass undisturbed until late in the spring, giving queen bumblebees safe places to rest, protect themselves from predators and for shelter at night and colder days.

## Methods

### Study Area

Harmonic radar field work took place between April 27, 2015 and May 15, 2015 at Rothamsted Research (Hertfordshire, UK, 51′48″13 N 0′22″8 W, Fig. 1a), with approval from the Rothamsted field experiment committee. The average daily minimum temperature over the period of radar tracking was 9.9 °C and the average daily maximum was 14.8 °C, and an average low of 6.3 °C with all periods of radar tracking occurring during part clouds/sun. Temperature data were collected from worldweatheronline.com. Wind speed and direction was obtained from COSMOS-UK^25^. Queen bumblebees were released and observed in the center of a hay meadow. This area was covered in short grass and normally grows wild flowers during the summer, but over the period of the study no wild flowers were observed. The closest tree cover was approximately 80 m away from the release site. There was an 8.6 ha patch of woodland 180 m away from the release site and residential gardens were within 150 m. The harmonic radar field site had no available sources of forage on the ground within the hay meadow surrounding the release site during our experiments so bees were not foraging during the times that no radar signals were detected within the hay meadow. Wild queen bumblebee observations were made between March 7, 2017 and March 15, 2017 in a woodland clearing (roughly 0.25 ha) in Epping Forest near South Woodford, UK (51′36″1 N 0′0″29 W), that was similar to the woodland close to the release site of radar monitored bees. Wild bumblebees were tracked in similar weather conditions as those for the harmonic radar observations – partly cloudy/sunny each day with an average minimum daily temperature of 8.7 °C and an average maximum daily temperature of 13.3 °C and an average low of 5.7 °C.

### Animals

Fertilized queens of *Bombus terrestris audax* were obtained directly from Biobest NV in Westerlo, Belgium, and transported at 4 °C to Rothamsted Research. Queens were kept at 4 °C in the dark until the start of the experiment. At the start of the radar monitoring experiments, queen bees were placed into small depressions in mounds of earth (5 cm high and 15 cm diameter) on the ground.

Tracking individual bees allowed us to see their patterns of flight and potential for dispersal over a reasonably lengthy period. However, individual tracking took two days per bee, both to track and to make sure that the bee had actually dispersed from the area. The simultaneous release of bees allowed us to gather much more data on the timing and direction of individual flights than could be obtained from individual releases, but at the cost of being unable to identify which individual produced each flight. Our intent was to gather data from queen bees early in the spring, during the time wild bees would be emerging from hibernation. The more bees we released individually, the greater the danger would be that one would remain in the tracking area and prevent us releasing further bees. Thus, it would have been difficult or impossible to collect individual tracking data from so many bees within the period that wild bees were emerging from hibernation. Judicious use of individual and mass tracking allowed us to maximize the data obtained during this period. Three bees were released individually, one on April 27, 2018 at 1:52 pm, a second on April 30 at 8:25 am, and a third on May 1, 2015 at 8:18 am. A group of 17 queen bumblebees were simultaneously released on May 11, 2015 at 10:14 am. These bees were placed in similar mounds arranged in a 4 × 4 grid whose sides were aligned with the principal compass directions with a single extra mound to the East. Mounds were separated from their nearest neighbors by 115 cm. Bumblebees hibernate below the soil-leaf litter interface, usually close to trees^26^. We could not exactly recreate these conditions since the radar transponders risked getting caught in the soil or leaf litter, so we placed the bees on mounds of loose earth. These also raised the bees above the level of the grass to ensure they would not become entangled while emerging from their hibernation state. The bees were placed in small depressions to ensure they would not accidentally fall off their mounds. The mound also gave the bee a recognizable site that she could learn and return to if she wanted.

### Radar tracking

The harmonic radar and tracking procedures are described in detail elsewhere^17,27^. Briefly, movements of queen bees were tracked using 32 mm harmonic radar. The radar was located at the Southeast edge of the experimental field where it rotated once every 3 s. Bees we wished to track were fitted with a transponder (consisting of a 16 mm vertical dipole) on their thorax. When activated by the radar beam, these transponders return the radar signal at half the original wavelength and twice the frequency. The radar unit has a second parabolic dish with a receiver tuned to this harmonic frequency allowing us to distinguish the transponder signal from reflections from other nearby objects. The radar returned distance and direction coordinates of the queen bees’ position every 3 s as long as the bees remained within a line-of-sight radius of approximately 800 m (accuracy ~±2.5 m). Transponders were attached to the thorax of each queen bee (Fig. 1a inset) using superglue (Loctite Power Flex Gel, Henkel Ltd., Hemel Hempstead, UK). The harmonic radar requires a line-of-sight between the radar and transponder, so can seldom detect bees on the ground and sometimes does not detect very low flight (below ~0.25 m) when there are obstacles between the bee and the radar. The trackable area of the field was defined by the trees and hedgerows at the edges. We monitored the movements of the three individually released bees for 5–11 hours per day and the 17 simultaneously released bees for 10–15 hours per day over 5 days apart from day 4 on which heavy rain forced us to turn off the radar after 4 hours.

### Analysis of radar data

The harmonic radar returned range and azimuth coordinates for every time a transponder was detected. Flight data were visualized by converting the radar coordinates of the bees’ positions to GPS coordinates using custom scripts written by JLW in MATLAB (Mathworks, Natick, MA, USA). The following variables were extracted from the radar tracks. Time in flight was the time during which the tracks showed the bee to be in motion. This excluded time that the bee was known to be stopped but also necessarily excludes time in which the bee was not detected by the radar but may have been in motion. A flight of a bumblebee was considered to be all sequential positional fixes of a bee that had no more than 6 seconds between each sequential pair of positional fixes. Time between flights was the time from the last radar coordinate of an individual bee’s flight and the first radar coordinate of that individual bee’s following flight. Of the 17 bees monitored simultaneously, data were used for time between flights only if the end of a flight and the beginning of another flight were within 5 meters of each other. Because our observations show that bees, likely by chance, did not tend to land very close to each other, 5 m is a distance that we could be sure included only the subsequent flights of the same bee and rejected any possible flight of a different nearby bee. Wind speed and direction were obtained every 30 mins from a weather station approximately 1250 m from the release site via COSMOS-UK^25^. Each bumblebee flight was paired with the weather readings taken closest in time to the start of that flight. All data processing, analyses and data figures were created using MATLAB (Mathworks, Natick, MA, USA).

### Observations in the wild

On March 7, 9 and 15 of 2017, 140 observations of queen bumblebees (*Bombus terretris*) were made within a grassy clearing in woodland in Epping Forest near South Woodford, UK. Although we could not be certain how many of these observations were of the same individual, given the large number of bees we saw (both focal bees and untracked individuals) and those disturbed simply by walking through the meadow, and the fact that many of the focal bees we observed flew well outside of the area we were monitoring, we are confident that a large majority of the bees were only observed once. We chose focal bees to observe at random from those we saw flying through the meadow. We observed each focal bumblebee until we lost track of her visually, which generally occurred when she flew out of the area through the woods or high up into the air too far and/or too fast for us to maintain visual tracking. We monitored the time each focal bee spent on the ground and in flight, and how far she flew if she landed again before we lost track of her. We measured the distance flown by placing an item next to the landing positions to be measured once we lost track of the bumblebee. Although we did not witness any queen bumblebee emerge from her hibernation, the timing of our observations was early in the year, less than a week after our first sighting of queens within the greater London area in 2017, indicating that these queens had very recently emerged. The fact that we saw no nest searching on the first days of observation and only three instances of a queen nest searching over a week later indicate that the majority of queens had not entered the nest-searching phase, so had likely recently emerged from hibernation. On 50 occasions, the ‘resting’ behavior of bees while on the ground was disturbed by an observer. Because we were unsure whether our actions caused these bees to stop resting and fly away, these observations were not included in our analysis of time between flights.

### Dispersal model

To investigate the dispersal rate of our queen bumblebees, we modelled dispersal distance over time using a random walk model. An individual’s overall flights are represented by a sequence of distinct, independent segments (line segments joining two sequential positional fixes), which lengths and orientations are drawn at random from distributions that were parameterized using the harmonic radar data. Flight directions were chosen at random from the distributions of observed flight directions (Fig. 4a). Flight lengths were taken to be the mean length of flights observed to occur in the selected flight direction (Fig. 4c). The number of flights taken per day was estimated from the average amount of time between flights by the three individually-monitored bees, since time between flights was hard to estimate from the simultaneously monitored bee data.

Foraging flights of worker bees with transponders have been shown to be slightly longer without transponders^28^, potentially affecting the accuracy of our flight length model parameters. Our transponders weigh approximately 15 mg which is less than 3% of the mass of a normal Bombus terrestris queen bumblebee (~800 mg^29^; n.b. workers can carry up to 90% of their body mass in nectar^30^). However, the durations of queen bee flights observed in the wild were similar to those found in bees with transponders (Fig. 1b), suggesting that the transponders did not have a large effect on the flight behavior of queens and demonstrating that the behaviors we observed – short periods of flight separated by long periods of inactivity – are not an artifact of the tracking technique.
